# Morphogenesis, Flowering, and Gene Expression of *Dendranthema grandiflorum* in Response to Shift in Light Quality of Night Interruption

**DOI:** 10.3390/ijms160716497

**Published:** 2015-07-21

**Authors:** Yoo Gyeong Park, Sowbiya Muneer, Byoung Ryong Jeong

**Affiliations:** 1 Institute of Agriculture and Life Science, Gyeongsang National University, Jinju 660-701, Korea; E-Mail: iuyiuy09@naver.com; 2 Division of Applied Life Science (BK21 Plus), Graduate School, Gyeongsang National University, Jinju 660-701, Korea; E-Mail: sobiyakhan126@gmail.com; 3 Research Institute of Life Science, Gyeongsang National University, Jinju 660-701, Korea

**Keywords:** anthesis, blue LED (light-emitting diode), chrysanthemum, flowering, light quality, photomorphogenesis, photoreceptor

## Abstract

The impact of shifts in the spectral quality of light on morphogenesis, flowering, and photoperiodic gene expression during exposure to light quality of night interruption (NI) was investigated in *Dendranthema grandiflorum*. The circadian rhythms of plants grown in a closed walk-in growth chamber were interrupted at night for a total of 4 h, using light-emitting diodes with an intensity of 10 μmol·m^−2^·s^−1^ PPF. The light quality of the NI was shifted from one wavelength to another after the first 2 h. Light treatments consisting of all possible pairings of blue (B), red (R), far-red (Fr), and white (W) light were tested. Plants in the NI treatment groups exposed to Fr light grew larger than plants in other treatment groups. Of plants in NI treatment groups, those in the NI-WB treatment grew the least. In addition, the impact of shifts in the light quality of NI on leaf expansion was greater in treatment groups exposed to a combination of either B and R or R and W light, regardless of their order of supply. Flowering was observed in the NI-RB, NI-FrR, NI-BFr, NI-FrB, NI-WB, NI-FrW, NI-WFr, NI-WR, and SD (short-day) treatments, and was especially promoted in the NI-BFr and NI-FrB treatments. In a combined shift treatment of B and R or B and W light, the NI concluded with B light (NI-RB and NI-WB) treatment induced flowering. The transcriptional factors *phyA*, *cry1* and *FTL* (*FLOWERING LOCUS T*) were positively affected, while *phyB* and *AFT* were negatively affected. In conclusion, morphogenesis, flowering, and transcriptional factors were all significantly affected either positively or negatively by shifts in the light quality of NI. The light quality of the first 2 h of NI affected neither morphogenesis nor flowering, while the light quality of the last 2 h of NI significantly affected both morphogenesis and flowering.

## 1. Introduction

Light signals regulate plant growth and development by sustaining environmental conditions [1]. To enhance growth and development, 24 h day/night plants develop an endogenous circadian clock [2]. This circadian clock is used to anticipate environmental changes and coordinate physiological and behavioral changes with environmental shifts [3]. Most flowering plants are severely dependent on light intensity patterns similar to their natural photoperiodic conditions to trigger normal biological functions [4,5,6,7]. However, photoperiodic conditions can be manipulated artificially to induce early flowering for commercial horticulture [8]. Manipulation of the photoperiod can also reduce production costs by reducing production times and improving the overall quality of the crop [9]. The photoperiod regulates growth and flowering in photoperiodic plants [10,11,12].

The use of artificial lighting at night (night interruption, NI) can regulate the flowering of photoperiodic species [13,14]. The NI breaks a long dark period to deliver photoperiodic lighting and thereby create modified long day (LD) conditions [15]. It has been reported that *Cymbidium aloifolium* photosynthesizes during 4 h NIs of photon fluence as low as 3–5 μmol·m^−2^·s^−1^ suggesting that the increased growth and earlier flowering times that occur in response to NI be attributed to an increase in net photosynthesis during this NI period [16]. Therefore, even NIs of low intensity are economical and effective means of controlling flowering in some species. The duration of the NI used in many studies [16,17,18,19,20,21,22,23,24,25] investigating flowering control was ≥4 h. However, a short (15 min) interruption of the long-night phase effectively controlled flowering in chrysanthemum plants that were grown under B light during their photoperiod [24].

It is well known that the effect of light quality on flowering regulation varies among plant species [24]. Many long-day plants (LDPs) flower most rapidly when the artificial light they are exposed to contains far-red (Fr) wavelengths, particularly when the artificial light is applied at the end of the photoperiod [26,27]. In SDPs, a night break with red (R) light effectively inhibits flowering, while flowering is promoted by subsequent exposure to Fr light [28]. This suggests that R/Fr reversible phytochromes may be involved in this response. The phytochrome photoreceptors mediate light quality perception, stem elongation, and flowering in photoperiodic plants [29]. Recent molecular and genetic investigations [30,31] have revealed that phytochromes are essential for photoperiodic flowering in rice (a facultative SDP). Loss-of-function of all phytochrome rice genes (in the *se5* mutant or *phyA phyB phyC* triple mutant) resulted in a deficient photoperiodic response and early flowering in short-day (SD) and long-day (LD) [30,31].

Flowering control by NIs presumably depends on the intensity, duration, and light quality of NI, as well as the plant species under consideration. Numerous papers have investigated the overall inhibitory effect of NIs on flowering when the NIs was timed to occur during the middle of the dark period. Although the effects of the duration and light quality of NIs were studied by Higuchi *et al.* [24] and Craig and Runkle [29], the effects of shifts in light quality of the NI have yet to be documented. Interestingly, Higuchi *et al.* [24] reported that in chrysanthemum grown with white light during photoperiod, flowering was observed under the SD interrupted in the middle of the dark period by a low intensity of blue (B) or far-red (Fr), while no flowering was observed under the same SD interrupted by red (R). Similar flowering responses were observed in our previous studies with chrysanthemum [32]. Therefore, we hypothesized that shifts in the light quality of low-intensity NI during the 4 h NI would affect flowering, either synergistically or antagonistically, and the light quality of the last 2 h of NI would have greater impact on flowering than the first 2 h of NI, as shown in a classical study on seed germination being affected by R and Fr shifting. In this study, we investigated the effect of shifts in the light quality of low-intensity NIs on not only flowering but also morphogenesis of *Dendranthema grandiflorum* “Gaya Yellow” (a qualitative SDP). In addition, the study examined the processes mediated by photoreceptors, with the goal of devising potential applications in floricultural crop production.

## 2. Results

### 2.1. Morphogenesis

**Figure 1:**
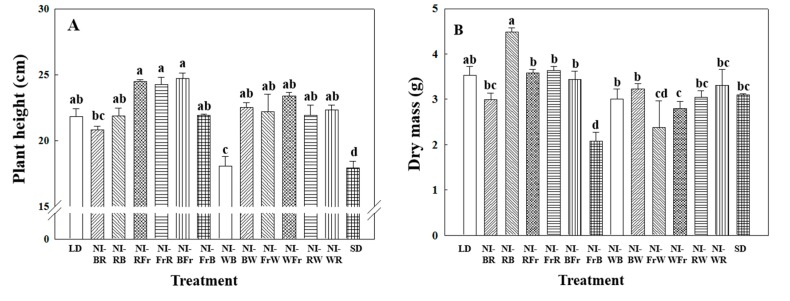

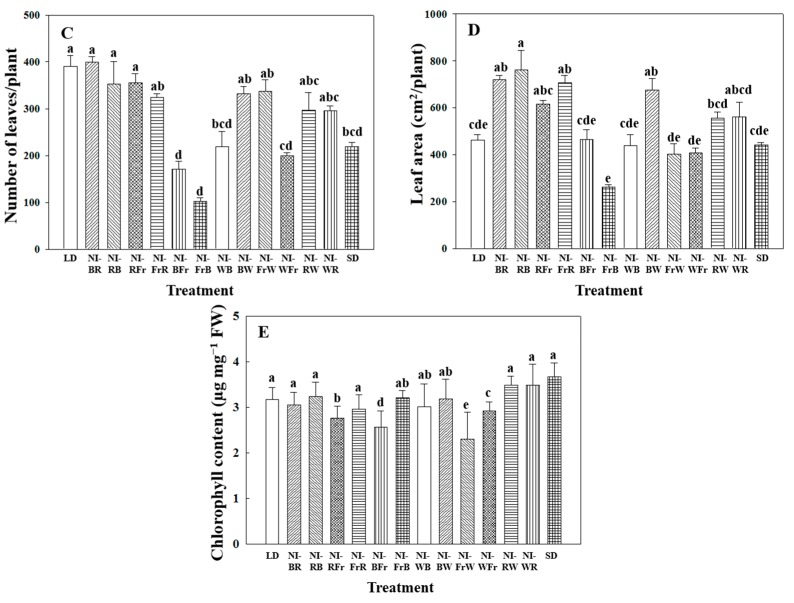
Effects of the shifts in the light quality of night interruption (NI) on (**A**) plant height; (**B**) dry mass; (**C**) Number of leaves per plant; (**D**) leaf area; and (**E**) chlorophyll content in *Dendranthema grandiflorum* “Gaya Yellow” (Please refer to Figure 1 for details of light quality of NI). Vertical bars indicate ± S.E of the means for *n* = 3. Means accompanied by different letters are significantly different (*p* < 0.05) according to the Tukey’s studentized range test.

Compared to the SD control, plant height increased in all NI treatments except the NI-WB treatment (Figure 1A). The height increases were greatest in NI treatment groups subjected to Fr light, regardless of the order of the two light qualities used. Dry masses were similar across all NI treatments to that of the SD control, with the exception of the LD, NI-RB, NI-FrB, and NR-FrW, treatments (Figure 1B). Compared to the SD control group, dry mass increased by 45% in the NI-RB treatment, and decreased by 33% in the NI-FrB treatment. The number of leaves per plant was significantly lower in the NI-BFr and NI-FrB treatments, compared to the SD control (Figure 1C). Leaf area was significantly higher in the NI-BR and NI-RB treatments, and lower in the NI-FrB treatments (Figure 1D), suggesting that leaf expansion was the result of the combined effect of the first and last light quality of NI. For example, the combination of the B and R light promotes leaf expansion, while the combination of the Fr and B suppresses it. Overall, the chlorophyll content was lower in the NI of Fr light treatment plants than that of plants in other treatment groups, regardless of the light quality of NI order used (Figure 1E). Chlorophyll content was 37% lower in the NI-FrW treatment compared to the SD control.

### 2.2. Flowering

**Figure 2:**
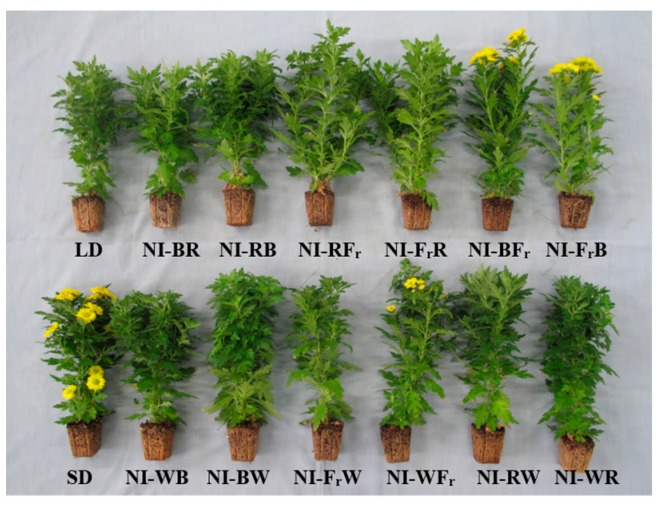

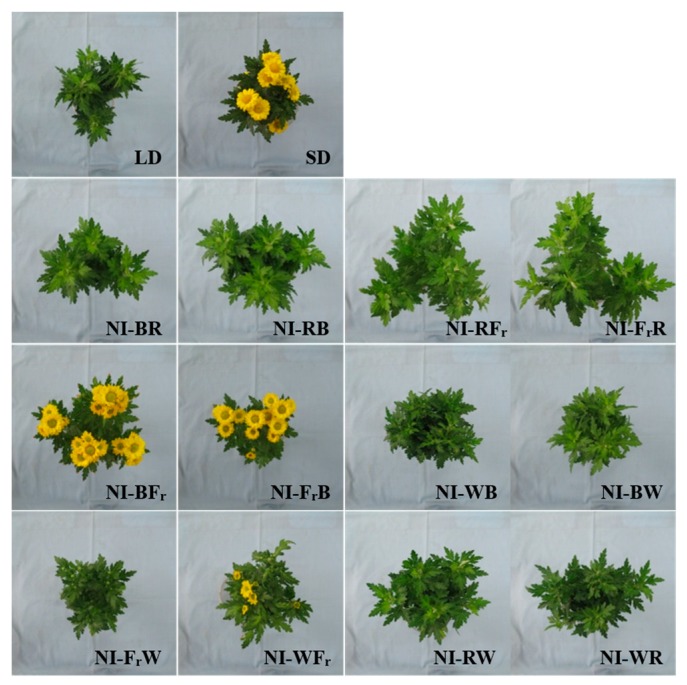
Effects of the shifts in the light quality of night interruption (NI) provided at 10 μmol·m^−2^·s^−1^ PPF (photosynthetic photon flux) on the flowering of *Dendranthema grandiflorum* “Gaya Yellow” measured at 49 days after treatment: (**A**) side view and (**B**) top view.

Flowering was induced by the NI-RB, NI-FrR, NI-BFr, NI-FrB, NI-WB, NI-FrW, NI-WFr, NI-WR, and SD treatments, and the DVB was shortened in the NI-BFr and NI-FrB treatments (Figure 2 and Table 1). The DVB increased in the NI-RB, NI-FrR, NI-FrW, and NI-WR treatments (Table 1). The results also showed that number of flowers per plant increased by 32% in the NI-BFr treatment, compared to the SD control (Table 1). The number of flowers per plant was the least in the NI-RB (Table 1).

**Table 1:** Effects of shifting the light quality of night interruption (NI) provided at 10 μmol·m^−2^·s^−1^ PPF (photosynthetic photon flux), on the flowering characteristics of *Dendranthema grandiflorum* “Gaya Yellow”, measured at 49 days after treatment.

Treatment	DVB ^y^ (Day)	Number of Flowers/Plant
LD	– ^z^	–
NI-BR	–	–
NI-RB	44.0	0.3 e ^w^
NI-RFr	–	–
NI-FrR	44.0	–
NI-BFr	14.8	27.3 a ^w^
NI-FrB	12.2	20.0 b ^w^
NI-WB	26.4	9.3 d ^w^
NI-BW	–	–
NI-FrW	33.7	12.3 c ^w^
NI-WFr	17.6	15.0 c ^w^
NI-RW	–	–
NI-WR	41.6	5.3 e ^w^
SD	13.6	20.6 b ^w^

^y^, Days from treatment initiation to visible flower bud, or days to visible buds; ^z^, No flowering; ^w^, Mean separation within columns by Tukey’s studentized range test at a 5% significance level.

### 2.3. Photoreceptor Gene Expression Analysis

**Figure 3:**
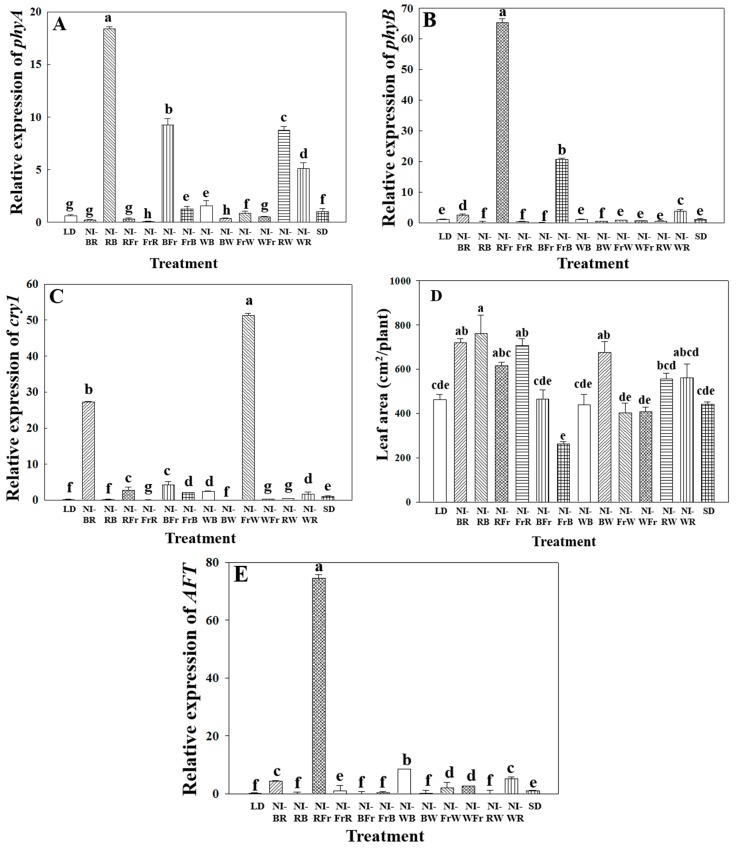
Relative gene expression of (**A**) *phyA* (**B**) *phyB*; (**C**) *cry1*; (**D**) *FTL*; and (**E**) *AFT* of *Dendranthema grandiflorum* “Gaya Yellow”. Vertical bars indicate ± S.E of the means for *n* = 3. Means accompanied by different letters are significantly different (*p* < 0.05) according to the Tukey’s studentized range test.

The results of the real-time PCR analysis indicated that *phyA* was highly expressed in the NI-RB, NI-BFr, NI-RW, and NI-WR treatments (Figure 3A). The relative expression rate of *phyB* was higher under the NI-RFr and NI-FrB treatments (Figure 3B). Higher expression rates of *cry1* were observed in the NI-BR and NI-FrW treatments, compared to the SD control (Figure 3C). The *FTL* and *AFT* genes were highly expressed in the NI-RFr as compared to the SD control (Figure 3D,E).

## 3. Discussion

### 3.1. Morphogenesis

Compared to the SD control, plant height decreased in the NI-WB treatment (Figure 1A). The successful use of exposure to B light (NI-WB) during the photoperiod to suppress plant elongation has previously been documented in chrysanthemum. Folta and Spalding [33] found that the inhibition response was associated with the involvement of cryptochromes and phototropins, and demonstrated that the inhibition process was a de-etiolated response. In this study, the suppression of vertical plant growth observed in the NI-WB treatment group was likely due to the use of B light in the second half of the NI. Moreover, this finding suggests that the B and W light receptors have a synergistic effect on the inhibition of vertical plant growth in chrysanthemum. The Fr light induced increase of the plant height, irrespective of the order of the two light qualities used (Figure 1A). The results are in agreement with those of Liao *et al.* [23] who suggested the enhancement of shoot extension by Fr light during the photoperiod. Shoot elongation is generally controlled by the endogenous gibberellins. The Fr light possesses the ability to induce the gibberellin biosynthesis resulting in increased shoot elongation [23].

The decrease of dry mass observed in the NI-FrB, NI-FrW, and NI-WFr treatment groups (Figure 1B) might be due to the effect of the Fr, as evidenced by the existence of the plant’s shade avoidance response, which is often accompanied by reductions in leaf area, shoot biomass, and the size of harvestable organs, a likely consequence of the reallocation of resources towards reproductive structures [34,35,36]. Compared to the SD control group, dry mass was significantly higher by 45% in the NI-RB treatment (Figure 1B) indicating that the treatment promoted photosynthesis, even at low light intensities. Photon fluxes lower than the compensation point still can help the plant growth by increasing the daily net photosynthesis, and the accumulative effect of this over the whole culture period could be considerable. Although the reason for this is not clear, similarly, significantly increased dry masses by the NI at 3–7 μmol·m^−2^·s^−1^ were reported in *Cymbidium* [16]. Also the NI with low photon fluxes promoted plant growth and flowering in *Cyclamen* (by 20 μmol·m^−2^·s^−1^ photon flux) [37], and Pansy and Hibiscus (by 40–50 μmol·m^−2^·s^−1^· photon flux) [38]. Daily light integral in this study was only 2.2% higher in the NI-RB (6.62 mol·m^−2^·day) than the SD control (6.48 mol·m^−2^·day). Therefore, in this study, the increase in the dry mass of plants in the NI-RB treatment group could be attributable to the synergistic effects of the yet unknown reasons of the R and B on vegetative growth characteristics such as leaf area which had a significant 73% increase in the NI-RB over the SD control.

The number of leaves per plant was significantly lower in the NI-BFr and NI-FrB treatments as compared to the SD control; this was likely due to the termination of vegetative growth caused by early flowering (Figure 1C). Constitutively, the number of leaves was dependent on changes in the number of days to flowering, especially when flowering was initiated early [12]. Decrease in chlorophyll content in the NI-FrW treatment as compared to the SD control in this study suggests that the Fr fluorescence consisted of low-intensity light as compared to the higher levels of incident light reflected from the leaves in this spectral region [39].

### 3.2. Flowering

The DVB (days from treatment initiation to visible flower bud) was higher in the NI-RB, NI-FrR, NI-FrW, and NI-WR treatments than the SD control (Table 1). This suggests that B, R, W, and Fr light receptors had an antagonistic effect on the promotion of flowering in chrysanthemum plants. The shortened DVBs in the NI-BFr and NI-FrB treatments (Table 1) suggest that in chrysanthemum (a SDP), flowering was promoted by the synergistic effects of B and Fr light. In a combined shifting treatment of B and R light or B and W light, the NI ending with B light (NI-RB and NI-WB) induced flowering. Thus, it is probable that the B light receptor can enhance floral-inducer activity, resulting in a higher energy requirement for the promotion of flowering. Previously, it was reported that Fr and B light promote flowering, whereas R light often inhibits it [12]. However, in both the combined R and Fr light treatment (NI-RFr and NI-FrR), and the Fr and W (NI-FrW and NI-WFr) light treatment, flowering was not affected by changes in the light quality of the NI. These findings suggest that the *P*_r_/*P*_fr_ ratio might have a bigger impact on flowering than the effects of changes in the light quality of the NI. In many SDPs, the NI is effective only when the supplied dose of light is sufficient to fully photoconvert all P_r_ (the phytochrome that absorbs R light) [40]. A subsequent exposure to Fr light, which photoconverts the pigment back to its physiologically inactive *P*_r_ form, restores the flowering response.

In this study, taller plants due to Fr light produced more flowers per plant, and this could be explained by two reasons. The increase in the number of flowers per plant in the NI-BFr light (Table 1) might be due firstly to high light energy induction and evasion of darkness in the shoot with longer internodes, which is known as the shade avoidance response. The reactions in response to shade avoidance are all initiated by a single environmental signal: the reduction in the ratio of R to Fr radiation (*i.e.*, R:Fr) that occurs within crowded plant communities [41]. This shade avoidance response is characterized by increased hypocotyl, stem, and petiole elongation; a more erect leaf position; increased apical dominance; and early flowering [42,43]. When grown in a close proximity to neighboring plants, many species grow elongated stems and smaller leaves, a behavior referred to as the shade avoidance response [44]. This response increases their ability to access sunlight by growing taller than other plants, and thus constitutes a competitive advantage [44]. This increase in the height growth could results in an increase in penetration of light energy through the canopy; leading to the increased production of photosynthetic assimilates during the photoperiod, due to increased stem growth and the development of a larger number of axillary buds. The second reason for the increased number of flowers in the NI-BFr light could be due to B light-related photoreceptors playing important roles toward inducing the flowering responses [45,46]. This finding on the increase in the number of flowers per plant in the NI-BFr light suggests that the B and Fr light, when given in this order, has a synergistic effect on the increase of the number of flowers per plant.

### 3.3. Photoreceptor Gene Expression Analysis

The *phyA* mediates the promotion of flowering by Fr light [47,48]. In contrast, *phyB* acts in a partially redundant manner with *phytochrome D* (*phyD*) and *phytochrome E* (*phyE*), mediating the inhibition of flowering by R light [34,49,50,51,52]. However, the function of *phyB* in floral initiation may be more complex than a simple floral inhibitor. The *cry1* and *cry2* act redundantly to mediate the promotion of flowering by B light [48,53]. Photoperiodic floral initiation is regulated by a systemic flowering inducer (florigen) and inhibitor (antiflorigen) such as the *FTL* and *AFT* genes, respectively, which are produced in the leaves [54].

The flowering that occurred in the NI-FrB treatment, even when the *phyB* gene (which is thought to be a flowering inhibitor gene) was expressed may be caused by high levels of expression of the flowering promotor genes *phyA* and *cry1* (Figure 3A–C). The *phyB*-mediated antiflorigen production system plays a predominant role in the obligate photoperiodic flowering response in chrysanthemum, allowing the strict maintenance of the vegetative state under non-inductive photoperiod conditions [54]. In the NI-RW treatment, even though a high level of *phyA* gene expression was induced, the plants did not flower (Figure 3A). Similarly, the lack of flowering was observed in the NI-BR treatment, in spite of high levels of *cry1* and *FTL* expression (Figure 3C,D). In this treatment, it is thought that although the first 2 h of NI with B light promoted flowering by stimulating the expression of the *cry1* and *FTL* genes, flowering was suppressed by exposure to R light during the last 2 h of the NI and/or interactions among the flowering-related genes. The absence of flowering that was observed even when the *FTL* gene was highly expressed in the NI-RFr treatment may be due to the high expression levels of the *phyB* and *AFT* genes observed in that treatment (Figure 3B,D,E). Based on the results in the NI-BR and NI-RFr treatment, it is assumed that the R light has relatively large, while the Fr light has relatively small, effect on flowering in chrysanthemum. The flowering-suppressing *AFT* gene was most highly expressed in the NI-RFr treatment, in which flowering was not observed as expected, and slightly expressed in the NI-WB treatment, in which plants did flower. It is assumed that R light has more effect on flowering in chrysanthemum than W light.

The overall expression patterns of photomorphogenic genes observed in this study are different from those observed in previous studies, as described in the previous paragraph. These differences might be due to the use of different chrysanthemum genotypes, differences in the experimental environment, or differences in sample collection time, the plant part from which the samples were collected, and the specific wavelengths of light produced by the LEDs used in each study. It seems contradictory to have similarly low levels of most photoreceptor genes in both inductive (SD) and noninductive (LD) treatments. The real reason for low expressions of most photoreceptor genes in both LD and SD treatments relative to the NI treatment is not clearly explainable. It could be that the expression level of these genes changes over time, presumably the greatest at the early stage of flowering induction and gradually decreasing afterward. Thus, it might be necessary to test whether ≤4 h of NI treatment with specific wavelengths and at considerably lower PPFs than currently employed can be used to suppress flowering in chrysanthemum, a qualitative SDP. The practical applicability of the light quality shifting of NI treatments in commercial crop production systems needs to be investigated in cases where the goal is the prevention of physiological disorders caused by light contamination during the dark period. In potted plants, consumers usually prefer smaller plant height. Therefore, B light has potential practical applicability for potted floricultural crops. We recommended that use in combination of B and Fr light (NI-BFr and NI-FrB) promote flowering and get many flowers, while use in combination of Fr and W light (NI-FrW) prolong flowering. In addition, the optimization of NI treatments in terms of intensity, duration, wavelength, and time of application also requires further study.

## 4. Experimental Section

### 4.1. Plant Material and Growth Conditions

Spray-type chrysanthemum (*Dendranthema grandiflorum* “Gaya Yellow” (a qualitative short-day plant, SDP)) cuttings, provided by the Flower Research Institute of the Gyeongsangnam-do Agricultural Research and Extension Services, Republic of Korea, were stuck in 50-cell plug trays containing a commercial medium (Tosilee Medium, Shinan Grow Co., Jinju, Korea), then placed on a glasshouse bench for rooting. Rooted cuttings were transferred to a closed walk-in growth chamber 7700 cm × 2500 cm × 2695 cm for 12 days at 20 ± 1 °C, 60% ± 10% RH, and 140 μmol·m^−2^·s^−1^ PPF provided by fluorescent lamps (F48T12-CW-VHO, Philips Co., Ltd., Eindhoven, The Netherlands), for acclimatization. This growth chamber was specially designed to blow in the conditioned air horizontally into the growing spaces through numerous uniformly distributed holes. The CO_2_ concentration was maintained at atmospheric levels (350 ± 50 μmol·mol^−1^) by supplementation from a compressed gas tank. The critical day length required for flowering in the SDP used in this study was about 14 h; therefore, a 10 h period of darkness was sufficient to initiate flowering. During the acclimatization period, plants were grown under a 16 h photoperiod (a long day condition, LD) to suppress flower initiation. After 12 days of acclimatization, the plants (approximately 13.3 cm in height) were transferred to the photoperiodic light treatments. The plants were fertilized once a day with a multipurpose greenhouse nutrient solution (in mg·L^−1^ Ca(NO_3_)_2_·4H_2_O 737.0, KNO_3_ 343.4, KH_2_PO_4_ 163.2, K_2_SO_4_ 43.5, MgSO_4_·H_2_O 246.0, NH_4_NO_3_ 80.0, Fe-EDTA 15.0, H_3_BO_3_ 1.40, NaMoO_4_·2H_2_O 0.12, MnSO_4_·4H_2_O 2.10, and ZnSO_4_·7H_2_O 0.44 (electrical conductivity 1.5 mS·cm^−1^) throughout the experiment.

### 4.2. Photoperiodic Light Treatments

**Figure 4:**
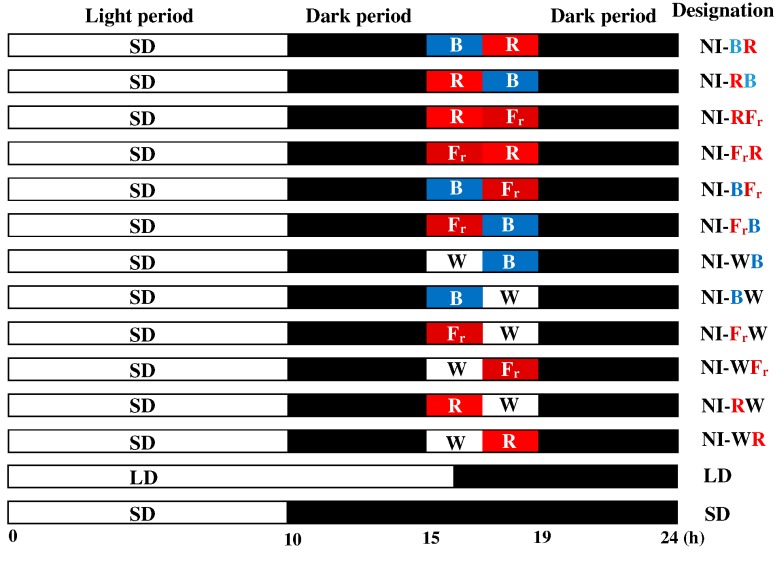
Representative image of the shifts in the light quality of night interruption (NI) by light emitting diodes (LEDs) during 4 h night interruptions (NI) of the 10 h short-day (SD) treatments in *Dendranthema grandiflorum* “Gaya Yellow”: NI-BR, blue to red; NI-RB, red to blue; NI-RFr, red to far-red; NI-FrR, far-red to red; NI-BFr, blue to far-red; NI-FrB, far-red to blue; NI-WB, white to blue; NI-BW, blue to white; NI-FrW, far-red to white; NI-WFr, white to far-red; NI-RW, red to white; and NI-WR, white to red. The LD indicates the 16 h long day treatment.

The plants were grown under 180 μmol·m^−2^·s^−1^ PPF provided by white (W) light-emitting diodes (LEDs) (MEF50120, More Electronics Co., Ltd., Changwon, Korea), under light treatment regimens of either a long day (LD, 16 h light/8 h dark), short-day (SD, 10 h light/14 h dark), or SD with a night interruption (NI) for a total of 4 h (from 11:00 p.m. to 3:00 a.m.), using LEDs at an intensity of 10 μmol·m^−2^·s^−1^ PPF. The photoperiod was started at 8:00 a.m. every day in all treatments. The light quality of the night interruption (NI) was shifted from one state to another after the first 2 h. Light quality combinations included all possible paired arrangements of blue (B, 450 nm), red (R, 660 nm), far-red (Fr, 730 nm), and white (W, 400–750 nm, with 28% B, 37% R, and 15% Fr light) light, as follows: from B to R (NI-BR), from R to B (NI-RB), from R to Fr (NI-RFr), from Fr to R (NI-FrR), from B to Fr (NI-BFr), from Fr to B (NI-FrB), from W to B (NI-WB), from B to W (NI-BW), from Fr to W (NI-FrW), from W to Fr (NI-WFr), from R to W (NI-RW), and from W to R (NI-WR) (Figure 4). All NI treatments were provided to plants separately, by subdividing the plant growth chamber with black cloth to prevent the spatial overlap of different light treatments. The LD and uninterrupted SD conditions were used as controls.

**Figure 5:**
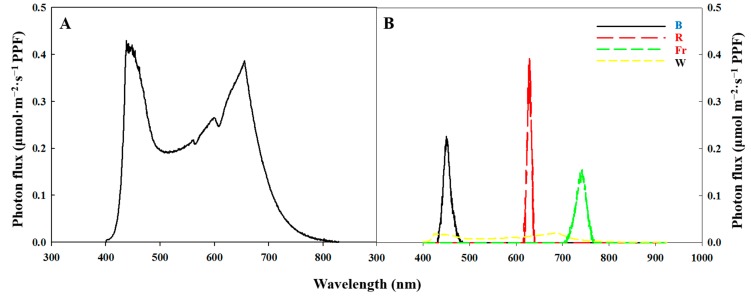
Spectral distribution of light used in a closed walk-in growth chamber: white light emitting diodes (LEDs) used as daily light (**A**) and different LEDs (B, blue; R, red; Fr, far-red; and W, white) used as light of night interruption (NI) (**B**).

The spectral distributions of all light treatments were scanned using a spectroradiometer (USB 2000 Fiber Optic Spectrometer, Ocean Optics Inc., Dunedin, FL, USA; wavelength detection range of 200 to 1000 nm) 25 cm from the bench top, at an interval of 1 nm (Figure 5). In each light treatment, the spectral distribution and the average value of maximum absolute irradiance were measured at three locations on the plant growing bench.

### 4.3. Data Collection and Analysis

The cultivation experiment with all the treatments was repeated twice and, since the results of both experiments showed same trends, only results of the second experiment were presented in this manuscript. At 49 days after the initiation of the photoperiodic treatments, the plant height, number of leaves per plant, chlorophyll content, fresh and dry masses of the shoot and root, percentage of flowering plants, days from treatment initiation to visible buds (DVB), number of flowers and flower buds per plant (mentioned as “number of flowers hereafter”), and the expression of important photoreceptor genes were either measured or assessed.

For the estimation of chlorophyll content, 10 mg of fresh leaf sample was taken from the young fully developed leaves from each biological replicate, and extracted using 80% ice-cold acetone. After centrifugation at 3000 rpm, the absorbance of the supernatant was measured using a spectrophotometer (Biochrom Libra S22, Biochrom Co., Ltd., Holliston, MA, USA) at 663 and 645 nm. Calculation of total chlorophyll was done according to the method described by Dere *et al.* [55]. The dry masses of the shoots and roots were determined after drying the samples in an oven (Model FO-450M, Jeio Technology Co., Ltd., Seoul, Korea) at 75 °C for 3 days.

### 4.4. Total RNA Isolation, cDNA Synthesis, and Real-Time Polymerase Chain Reaction (PCR) of Selected Genes

For total RNA extraction, the most recently mature leaf was used which was collected in the morning (9:00 a.m.) at 30 days after the initiation of the photoperiodic light treatments in all treatments when the plants in the SD treatment already started to bloom, those in the inductive NI treatments had flower buds formed, and those in the LD treatment were still vegetative. Total RNA was extracted using an RNA isolation kit according to the manufacturer’s instructions (Promega, Madison, WI, USA). The sample collection time (9:00 a.m.) was selected because photosynthetic rates are high, and all plant processes including gene expression are very active at this point of time. One microgram of DNase-treated RNA was reverse transcribed using a reverse transcription kit (Promega, Madison, WI, USA) to synthesize first-strand cDNA, which was used as a template for the polymerase chain reaction (PCR). Real-time PCR was performed using a Rotor-Gene Q 2plex HRM Platform (Rotor-Gene Q 2plex HRM Platform, Westburg, The Netherland) and SYBR green from a Qiagen qPCR kit (Qiagen OneStep RT-PCR Kit, Westburg, The Netherland) as a reference dye. Independent real-time PCR reactions with equal amounts of cDNA were performed using the *phytochrome A* (*phyA*), *phytochrome B* (*phyB*), *cryptochrome 1* (*cry1*), *Anti-florigenic FT/TFL1* family protein (*AFT*), and *FLOWERING LOCUS T* (*FTL*) gene primers (the primer sequences are shown in Table 2). *Actin* was used as an internal control, as it is commonly used to normalize molecular expression studies because of its high level of conservation as an endogenous housekeeping gene. The real-time PCR conditions were as follows: initial denaturation for 5 min at 95 °C, followed by 25 cycles consisting of 20 s at 95 °C, 30 s at 60 °C, and 30 s at 72 °C, then 10 min at 72 °C for final extension. To calculate the relative expression levels (treatment/control, SD), the individual expression values of treatment plants were divided by the mean value for SDPs at each sampling date.

**Table 2:** List of primers used to quantify gene expression levels.

Gene	Accession Number	Forward Primer	Reverse Primer
*Ch-phyA*	EU915082	5′-GACAGTGTCAGGCTTCAACAAG-3′	5′-ACCACCAGTGTGTGTTATCCTG-3′
*Ch-phyB*	NM_127435	5′-GTGCTAGGGAGATTACGCTTTC-3′	5′-CCAGCTTCTGAGACTGAACAGA-3′
*Ch-* *cry1*	NM_116961	5′-CGTAAGGGATCACCGAGTAAAG-3′	5′-CTTTTAGGTGGGAGTTGTGGAG-3′
*Ch-* *FTL*	AB839767	5′-ACAACGGACTCCTCATTTGG-3′	5′-CGCGAAACTACGAGTGTTGA-3′
*Ch-* *AFT*	AB839766	5′-AGAACACCTCCATTGGATCG-3′	5′-CTGGAACTAGGTGGCCTCAC-3′
*Ch-* *Actin*	AB205087	5′-CGTTTGGATCTTGCTGGTCG-3′	5′-CAGGACATCTGAAACGCTCA-3′

### 4.5. Statistical Analysis

A randomized complete block design with 3 replicates of 2 plants each was used in this experiment. The data collected during the experiment were analyzed for statistical significance using the SAS (Statistical Analysis System, V. 9.1, Cary, NC, USA) program. Differences among the treatment means were assessed using Tukey’s studentized range test at *p* < 0.05. Graphing was performed with Sigma Plot 10.0 (Systat Software, Inc., San Jose, CA, USA).

## 5. Conclusions

Plant height was the smallest in the NI-WB treatment among all NI treatments. The impact of changing the light quality at low intensity NI treatments on leaf expansion was greater in treatments using a combination of B and R or R and W light, regardless of their order of use. Flowering was observed in the NI-RB, NI-FrR, NI-BFr, NI-FrB, NI-WB, NI-FrW, NI-WFr, NI-WR, and SD treatments, and was especially promoted in the NI-BFr and NI-FrB treatments. In a combined shifting treatment of either B and R light or B and W light, the NI concluding with B light (NI-RB and NI-WB) exposure induced flowering. Thus, it is likely that the B light receptor enhances the activity of floral inducers, resulting in a higher energy requirement for the promotion of flowering. The transcriptional factors *phyA*, *cry1*, and *FTL* were positively affected, while *phyB* and *AFT* were negatively affected. The expression of morphogenesis, flowering, and transcriptional factors was significantly affected either positively or negatively by changes in the light quality of the NI. Statistically, the light quality of the first 2 h of NI exposure affected neither morphogenesis nor flowering, while the light quality of the last 2 h of NI exposure significantly affected both morphogenesis and flowering, as we hypothesized. Further studies are required to investigate the effects of B light supplementation on flowering promotion, photoreceptor gene expression, and protein production in other cultivars of chrysanthemum.

## Abbreviations

*AFT* (*Anti-florigenic FT/TFL1* family protein); B (blue); *cry1* (*cryptochrome 1*); DVB (days after treatment initiation to visible flower bud or days to visible buds); *FTL* (*FLOWERING LOCUS T*); Fr (far-red); LD (long day); LDP (long day plant); LEDs (light emitting diodes); NI (night interruption); PCR (polymerase chain reaction); *phyA* (*phytochrome A*); *phy**B* (*phytochrome* *B*); *phy**C* (*phytochrome* *C*); *phy**D* (*phytochrome* *D*); *phy**E* (*phytochrome* *E*); R (red); SD (short-day); SDP (short-day plant); W (white).
